# A Medical Mrs. Grundy

**Published:** 1898-06-25

**Authors:** 


### A MEDICAL MRS. GRUNDY.

A curious little association has been started, having for one of its objects the provision of what it calls "expert" medical advice to certain people, the nominees of medical practitioners, at less than the ordinary rate. Although the medical support which it has received is too unimportant to induce us to take it seriously as an indication of the way the wind blows, it does suggest that there is just a little breeze, a little flutter which aims at disturbing the tranquillity of that respectable institution the two-guinea fee. What is maintained by its founders is the fairly obvious verity that some of the abuse which exists in the out-patient departments of our hospitals is the direct outcome of the fact that the private fees of consultants are so high. The truth of this view of the matter is fully admitted by many medical men who have studied the question; and, indeed, our contemporary, the *Lancet*, goes so far as to say that "no person, and especially no medical practitioner, can fail to be struck with the painful difficulty of getting expert opinions for those of modest circumstances, and especially of getting surgical work done for them, except at prohibitive prices which suggest or compel application to a hospital." The general practitioner has to accommodate his fees to the means of his patient; but the fee of the consultant or the "expert," as he seems now to be called, remains, in theory at least, the same for all, and certainly the time is coming, if it has not already come, for some modification of this cast-iron rule. Either leaders should go up, or the juniors should be allowed to come down. There is a certain inherent absurdity in a man giving a couple of hundred consultations a week at a hospital, free to all who come, and then maintaining that it is impossible for him to see a patient at his own house for less than two guineas. Nor is it true. Every one knows quite well that London specialists are kindly-hearted men; and that, although a stranger going to see any of them without an introduction, without a piteous tale, and without a humiliating petition to be let off, will have to pay his two guineas, yet the crowds in the waiting-rooms (where there are crowds) are much "watered" by patients who are seen for nothing—governesses, clergy, upper servants, and other protéges who could, and often would, pay half a guinea if the dignity of the consultant would allow it, and also by people who, as a fact, pay even less than half a guinea, coming as they do two or three times for each fee paid. All this is done that the physician may not lose caste, and may be able to hold his head up before his fellows as one who never takes less than the proper fee. But does it not seem a little bit of a subterfuge, and is it altogether open and above board? Of course, a man would be laughed to scorn who would suggest that a consultant might without loss of dignity accept five shillings for his fee. Nevertheless, it is impossible to shut one's eyes to the very simple fact that professional leaders find it quite possible to see and investigate a dozen cases in the morning, and that a dozen times five shillings in a morning means nearly a thousand a year. How many junior consultants are there not who would be thankful for half that income? The oddity of the position is that it is as cheap to go to the man at the top of the tree as to the man at the bottom, and that, in consequence of this ingenious arrangement, the latter is left with nothing to do except at the hospital. A sort of medical Mrs. Grundy forbids the young man to take less than "the proper fee," while a sort of hospital Mrs. Grundy forbids the hospitals to take anything at all. How, then, are those who are neither poor nor rich to obtain that "expert," or, as it is commonly called, specialist opinion which is open to those at both extremes of the social scale? Clearly a great premium is thus set upon hospital abuse, and these various Mrs. Grundys have much to answer for.

